# What is the quality of life in patients with long COVID compared to a healthy control group?

**DOI:** 10.3389/fpubh.2022.975992

**Published:** 2022-11-02

**Authors:** Dávid Líška, Erika Liptaková, Adriana Babičová, Ladislav Batalik, Patrícia Shtin Baňárová, Silvia Dobrodenková

**Affiliations:** ^1^Department of Physical Education and Sports, Faculty of Arts, Matej Bel University, Banská Bystrica, Slovakia; ^2^Technical University of Košice, Faculty of Economics, Košice, Slovakia; ^3^Children's Club for Disabled Children and Youngsters, Košice, Slovakia; ^4^Department of Rehabilitation, University Hospital Brno, Brno, Czechia; ^5^Department of Public Health, Faculty of Medicine, Masaryk University, Brno, Czechia; ^6^Faculty of Healthcare, Alexander Dubček University of Trenčín, Trenčín, Slovakia; ^7^Travel Health Clinic, Bratislava, Slovakia

**Keywords:** quality of life, long COVID, chronic fatigue, persistent symptoms, COVID-19

## Abstract

**Introduction:**

Many patients have prolonged symptoms after COVID-19 infection, which can affect patient quality of life (QOL). The aim of this study is to determine the quality of life in patients with long COVID, compared with healthy controls.

**Material and methods:**

The study was a prospective cross-sectional study using an anonymous online survey. The SF-36 questionnaire was chosen for quality of life measurement. The survey was distributed through the Facebook social media platform targeting groups of patients with long COVID. The control group was made up of physiotherapy and physical education students.

**Results:**

There was a significant difference in physical function, with a mean score of 94.9 (±9.4) among the students, compared to long COVID patients with a mean score of 66.2 (±25.4) (*p* < 0.001). A similar result was found in the physical role (*p* < 0.001). The overall quality of life score for college students was 578.0 (±111.9), and the overall score for patients with long COVID was 331.9 (±126.9).

**Conclusions:**

Patients with long COVID had a lower quality of life compared to the healthy control group, and this was associated with the negative effect of long-COVID. Lower quality of life in patients with long COVID is an important therapeutic goal, which requires attention.

## Introduction

Coronavirus disease 2019 (COVID-19) is an infectious disease caused by SARS CoV-2 virus. The disease was first diagnosed in Wuhan, China (1). SARS-CoV-2 (COVID-19) infection caused a major pandemic, leading to significant morbidity and mortality worldwide (2). Approximately 80% of affected patients have mild to moderate disease, and 5% of patients with severe disease developed critical disease (3). The clinical manifestation of COVID-19 has been shown to vary considerably, often with respiratory complications as the main symptoms (4). SARS-CoV-2 is notable for the fact that many patients develop persistent or new symptoms that last weeks or months; this is called long COVID (5, 6). Long COVID can also occur in children (7–10). The term “long COVID” is used to describe the presence of various symptoms lasting weeks or months after overcoming SARS-CoV-2 infection, regardless of viral status (11, 12). Long COVID can be continuous or recurrent (13).

Most patients with long COVID syndrome are negative for polymerase chain reaction (PCR), suggesting microbiological recovery (14). Similarly, most patients with long COVID show biochemical and radiological recovery. The mechanisms underlying long COVID are still largely unknown (15). The risk factors for long COVID-19 are ambiguous, but several potential risk factors have been identified (16). The risk of long COVID is twice as common in women as in men (13). Increasing age is a risk factor. The presence of more than five symptoms in the acute stage of the disease is associated with an increased risk of developing long COVID (17). The presence of comorbidities also increases the risk of developing disease.

Persistent symptoms after overcoming COVID-19 include fatigue, respiratory cardiovascular problems, musculoskeletal problems, neurological symptoms, and hematological, immunological, renal and gastrointestinal symptoms (18–25). These symptoms can significantly affect quality of life.

Quality of life (QOL) is defined as an individual's sense of well-being in terms of satisfaction with important aspects of life (26). QOL is a multidimensional concept that encompasses domains related to physical, mental, social, and emotional functioning. It is defined by the World Health Organization (WHO) as an individual's perception of their position in life in the context of the culture and value systems in which they live, and in relation to their goals, expectations, standards, and concerns (27). The concept of health-related quality of life (HRQOL) includes aspects of overall quality of life that can be proven to affect health, whether physical or mental. At the community level, HRQOL includes resources, conditions, policies, and practices at the community level that affect the perception of health and functional status in the population. Evaluating the functional status of patients is an important process, and measuring patient quality of life can lead to the objectification of their health status. Patients with long COVID often have multiple symptoms that can adversely affect quality of life. The purpose of this study is therefore to determine the quality of life in patients with long COVID compared to healthy controls.

## Materials and methods

The study was a prospective, cross-sectional study using an anonymous online survey. The survey was distributed through Facebook's social media platforms, targeting groups of patients with long COVID. The control group consisted of healthy individuals; therefore, students in physiotherapy and physical education were included in the study, for whom is assumption of a healthy group. Students from four universities were included in the control: (1) Matej Bel University, Department of Physical Education and Sports; (2) Slovak Medical University, Faculty of Healthcare Banská Bystrica; (3) Alexander Dubček University of Trenčín, Faculty of Healthcare; and (4) Technical University, Košice Department of Academic Sports. The questionnaire was distributed through internal online school systems.

This cross-sectional study was conducted between February 2022 and May 2022. Patients and students over the age of 18 years were included in the study. Students with visual impairment were not included in the study. After their informed consent was obtained, patients and students completed the structured Quality of Life (SF-36) questionnaire. They were asked for baseline data such as age, weight, and height. The quality of life of patients with long COVID was compared with the quality of life of the college students. Questions focusing on patient symptoms were added. Another added question was, “Compared to your pre-COVID-19 condition, how would you rate your health in general now?”

All procedures in this study involving human participants were in accordance with the ethical standards of the institutional and national research committee, and with the 1964 Helsinki Declaration and its later amendments, or comparable ethical standards. Informed consent was obtained from all individual participants involved in the study. The study was approved by the Ethics Committee of Matej Bel University under the number FF/123/2022.

### SF-36 questionnaire

The SF-36 questionnaire was chosen for the quality of life measurement. The SF-36-item questionnaire is a popular tool for assessing health-related quality of life (28–30). It includes eight health concepts: physical functioning, physical role, bodily pain, general health, vitality, social functioning, emotional role, and mental health (31). SF36 scores between 0 and 100 were assigned to each domain, with higher scores indicating a more favorable functional status. A higher SF36 score indicates a better health state: the maximum score (the best answer) for one item was 100, the minimum score (the worst answer) for one item was 0.

### The sample

**Figure 1:**
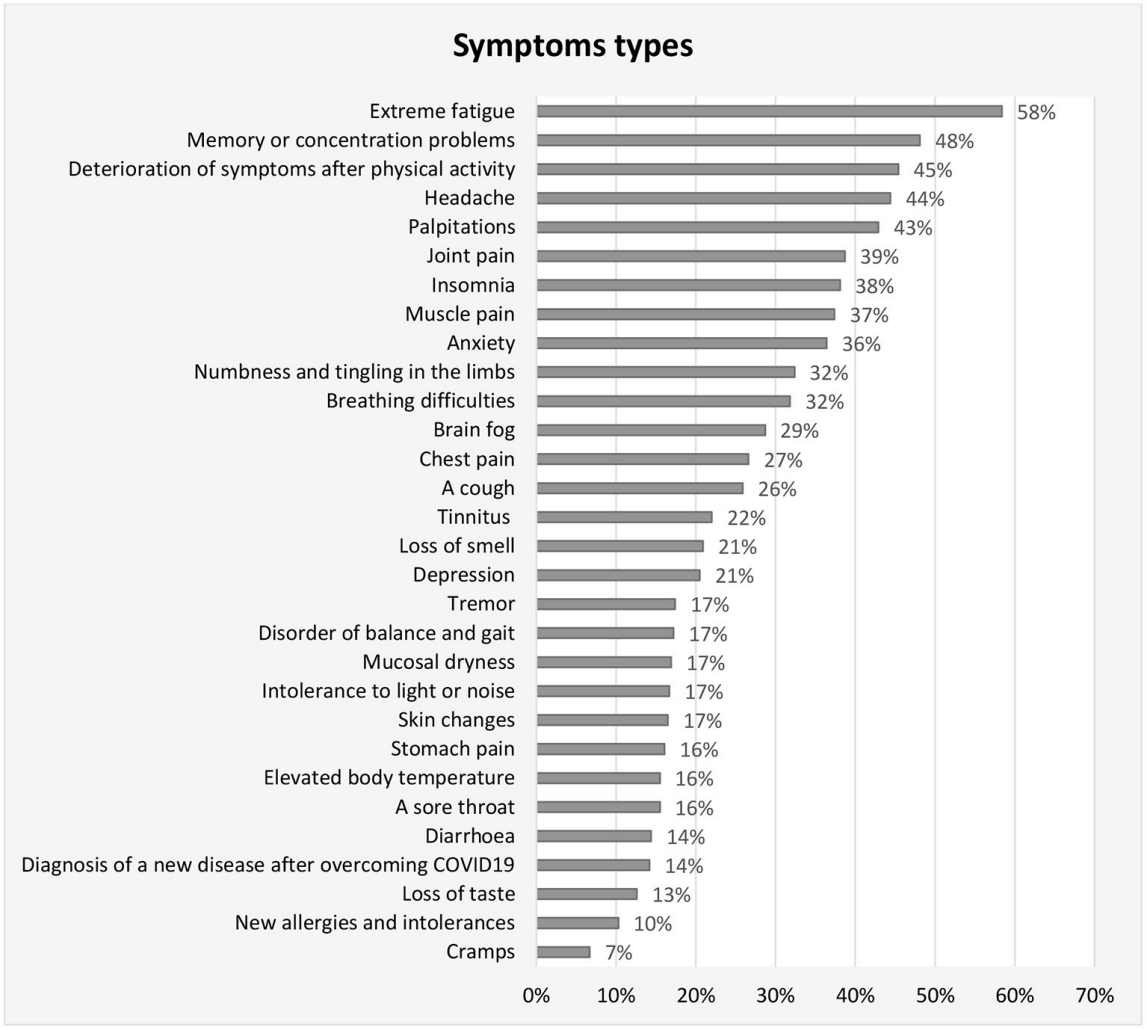
Types of symptoms among patients with long COVID.

The participants consisted of patients with long COVID (*n* = 469), control group consisted of college students (*n* = 338). Baseline characteristics are shown in Table 1. The most frequent symptoms were fatigue (58%), memory or concentration problems (48%), deterioration of symptoms after physical activity (45%), headache (44%), and palpitations (43%). The symptoms of the patients are shown in Figure 1.

**Table 1:** Baseline characteristics of the long COVID group and control group.

	**Long COVID patients**	**College students**
	**(*n* = 469)**	**(*n* = 338)**
Female	393 (83.7%)	220 (65.1%)
		
Age (years)	41.3 (± 10.5)	24.1 (± 6.9)
Men	37.3 (± 10.8)	26.5 (± 8.9)
Women	42.1 (± 10.3)	22.8 (± 4.9)
BMI	26.44 (± 6.2)	22.8 (± 3.4)
Men	27.2 (± 4.9)	24.8 (± 3.1)
Women	26.3 (± 6.3)	21.7 (± 3.0)
Height (cm)	169.3 (± 9.8)	172.2 (± 8.9)
Men	180.6 (± 15.6)	181.1 (± 6.5)
Women	167.0 (± 6.1)	197.4 (± 5.9)
Weight (kg)	76.2 (± 19.8)	68.1 (± 14.2)
Men	90.7 (± 19.1)	81.3 (± 11.7)
Women	73.4 (± 18.6)	60.9 (± 9.6)

### Statistical analysis

The results were uploaded to an Excel spreadsheet and subjected to statistical analysis using IBM SPSS Statistics 22.0 (IBM Corp. Chicago, IL, USA) and the SAS Enterprise Guide. Raw scale scores were calculated and then transformed according to the instructions in the SF-36 Health Survey Manual and Interpretation Guide. Distribution analysis was used to compare the distributions of the total scores between the group of patients and the group of students. Due to the non-normal distribution of scores, the non-parametric Mann-Whitney U test was used to find differences between the group of patients and the group of students in the eight domains of SF-36, and also in the total score. The level of significance was set at 0.05.

## Results

**Figure 2:**
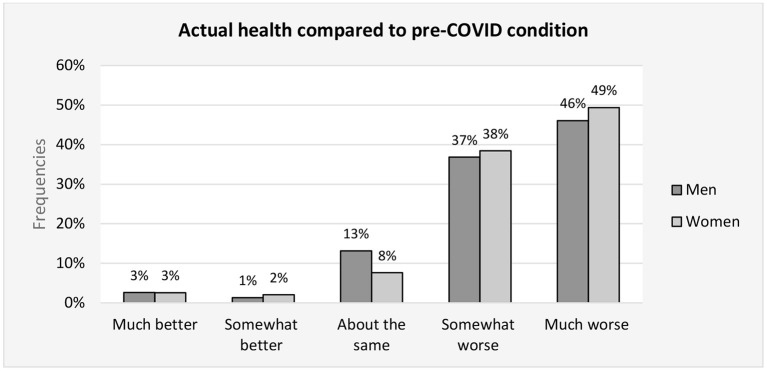
Actual health compared to pre-COVID condition - frequencies of patient answer.

Compared to pre-infection, 38.2% patients (36.8% men, 38.4% women) reported being in much worse condition (48.8% patients, 46.1% men, women 49.4%), the same condition 8.5% patients (13.2% men, 2 % women), better condition 1.9% patients (men 1.3%, women 2.0%), and Figure 2 much better condition 2.6% patients (2.6% men, 2.5% women).

**Figure 3:**
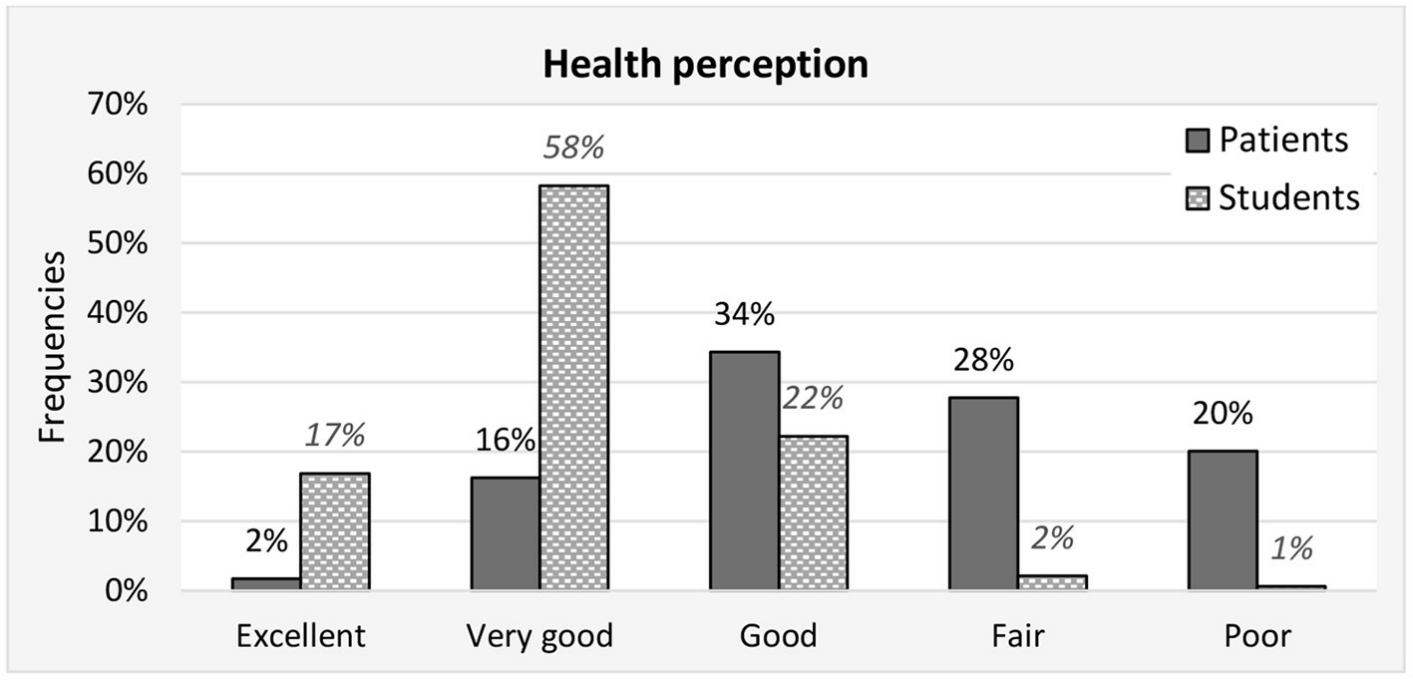
Health perception **-** frequencies of answers for patient and student groups.

Figure 3 shows perceptions of health. Seventy percent of students reported having an excellent or very good perception of health, but only 18% of patients. Conversely, up to 48% of the patients reported that they had a fair or poor perception of health, and only 2.7% of students. There is a statistically significant difference in health perception between the group of patients and group of students (Pearson Chi-square = 307.3; *p* < 0.001).

There was a significant difference in physical function between the long COVID patients, who had a mean score of 66.2 (±25.4), and students with a mean score of 94.9 (±9.4) (*p* < 0.001) A similar result was found in the role physical (*p* < 0.001). The incidence of pain in patients with long COVID was significantly higher than in the group of college students (*p* < 0.001). The mean general health score was 35.8 (±16.1) for the patients, and 58.6 (±12.9) for the students. There was a significant difference between the groups (*p* < 0.001). The mean vitality score for the patients was 29.8 (±18.3), and for college students 54.2 (±19.7). There was a significant difference between the groups (*p* < 0.001). The mean social functioning score was 34.8 (±20.8) for the patients and 75.9 (±21.8) for the students. The result was significant in favor of the college students (*p* < 0.001). There was a significant difference in favor of college students in the subcategory of emotional and mental health (*p* < 0.001). The overall quality of life score for college students was 578.0 (±111.9), and the overall score for patients with long COVID was 331.9 (±126.9). Table 2 gives the detailed results.

**Table 2:** Comparison between group of students and group of patients with long COVID in eight domains of the SF-36 questionnaire.

**SF-36 domains**	**Group**	**Mean Score**	**SD**	Z^*^	***p* Value**
Physical function	Students	94.9	9.4	18.58	< 0.001
	Patients	66.2	25.4		
Role limitations (physical)	Students	80.2	19.9	21.00	< 0.001
	Patients	34.1	21.4		
Bodily pain	Students	71.0	25.3	16.21	< 0.001
	Patients	39.4	19.2		
General health	Students	58.6	12.9	17.52	< 0.001
	Patients	35.8	16.1		
Vitality	Students	54.2	19.7	15.40	< 0.001
	Patients	29.8	18.3		
Social functioning	Students	75.9	21.8	19.58	< 0.001
	Patients	34.8	20.8		
Role limitations (emotional)	Students	77.0	23.4	11.23	< 0.001
	Patients	53.2	29.6		
Mental health	Students	66.4	18.2	17.77	< 0.001
	Patients	38.6	16.0		

* Z represents z-score calculated in Mann-Whitney U-test.

The score for each domain is given on a scale 0–100 where s score of 100 is the best answer, and a score of 0 is the worst answer.

**Figure 4:**
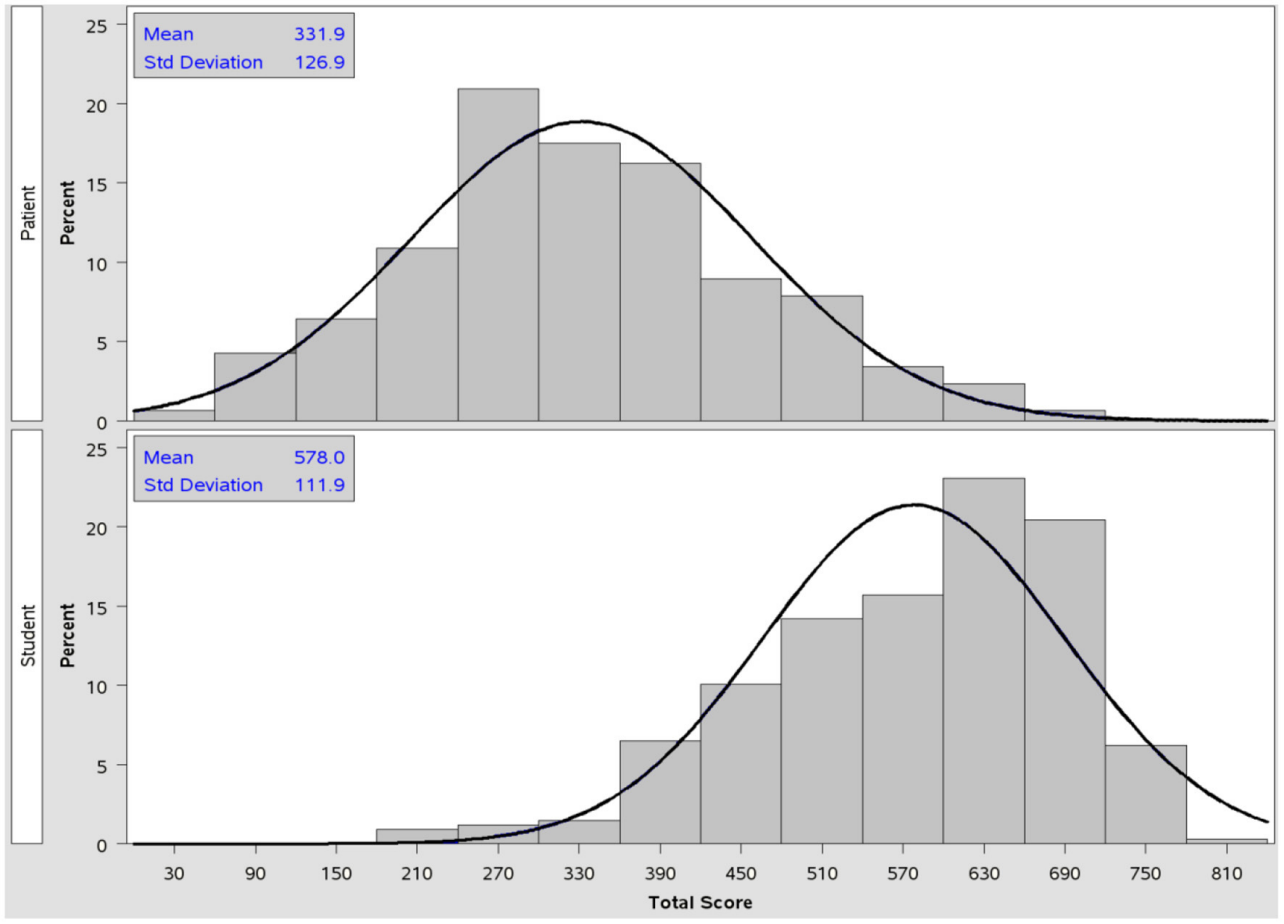
Comparison of the distributions of the SF-36 total score - patient group (upper picture) and student group (lower picture). Maximum possible total score is 800 (the best perception of each item in the questionnaire). Normality test results (Shapiro-Wilk test): group of patients (W = 0.9934; p = 0.0385), group of students (W = 0.9588; p < 0.001).

Figure 4 shows the distributions of total scores separately for the group of patients and group of students. The mean value of the total SF-36 score for the group of patients was 331.9 (±126.9), and 578.0 (±111.9) for the group of students. The variability of the total score was approximately the same in the compared groups, but mean values were statistically significant different (Z = 20.32; *p* < 0.001).

## Discussion

The main aim of our study was to point out the difficult conditions affecting patients with long COVID in terms of quality of life. Long COVID is a public health problem that needs to be defined, quantified, and described. We observed a reduction in quality of life in all parameters monitored in our study, compared to a healthy control group. The control group was chosen to show that patients with long COVID suffer from a lower quality of life. In our country, this serious diagnosis is often undervalued, and it is therefore important for us to point out that quality of life is significantly lower in patients with long COVID compared to a control group.

The long-term symptoms of COVID-19 can have a devastating effect (4, 5). Persistent symptoms in patients with long COVID lead to a reduction in patient quality of life. The subjective persistence of symptoms such as fatigue in patients can affect aspects of quality of life such as physical function, bodily pain, vitality, emotional health, and social functioning, which were significantly lower in patients compared to a healthy control group. Patients experienced a significant reduction in social functioning, which may indicate a reduced interest in engaging in normal life as a result of the disease. The social distancing of patients with long COVID can significantly affect their quality of life.

Chronic fatigue is a common manifestation of long COVID, and is also frequently reported after overcoming COVID-19. The fatigue experienced is greater than normal post-exercise fatigue, however; it is a constant state of exhaustion that reduces a person's energy, motivation, and concentration. This can have a significant effect on QOL. The incidence of patient fatigue in our study was significantly higher compared to the healthy control group. Chronic fatigue can adversely affect all aspects of quality of life (32).

Another symptom that can negatively contribute to poorer quality of life in patients is “brain fog.” Brain fog is characterized by long-lasting altered attention or cognitive function (33–36). Brain fog is a general term used to describe a feeling of cognitive impairment and impairment in the ability to concentrate. It affects a person's ability to think or concentrate. Cognitive impairment is associated with lower occupational function (37). A patient's inability to work and return to normal life can be exacerbated by a lower quality of life. Patients have experienced the appearance of several of the symptoms shown in Figure 1. The appearance of these symptoms can negatively affect quality of life. Dyspnoea is also common. A cough, chest pain, and headaches are other common symptoms that can adversely affect a patient's quality of life. Another symptom that can negatively affect QOL is sleep problems (38).

Most publications on COVID-19 and mental health have highlighted specific responses to the pandemic, such as anxiety, stress, and conditions related to altered routines, loneliness, and social isolation in uninfected individuals (39–41). Increased stress may remain in patients even after infection is overcome, and, as is well known, stress negatively affects quality of life (42–46). Psychological stressors are a public health problem. The body's response to stress is attributed to the activity of several axes, such as the hypothalamic-pituitary-adrenal (HPA) axis and the sympathetic adrenomedullary circuits (44, 47). Stressful life events, including the defeat of the SARS-CoV-2 virus, can affect the function of the neuroendocrine and immune systems, physical and mental wellbeing, and thus quality of life.

Stressful life events are indirectly related to quality of life and wellbeing (46–49). Pandemics, social isolation, and infection could be perceived in patients as very stressful life events, negatively affecting their quality of life. Posttraumatic stress disorder is known to occur after negative life experiences, and contribute to a lower quality of life (50, 51).

Long COVID is difficult to detect with common diagnostic tools and parameters, such as laboratory values (52). Another negative factor that may contribute to a lower quality of life in patients with long COVID is thus people's perceptions of patients around them. Patient symptoms are often simplified due to the absence of clear diagnostic methods. Trivialisation of symptoms by the environment, as well as by medical staff, can lead to an increase in anxiety and reduce overall quality of life in patients with long COVID.

Our study found that patients with long-COVID had a lower quality of life. This indicates a need for improvement quality of life in patients with long-COVID. It is necessary to examine different methods of treatment in order to improve the health status and quality of life of patients. The absence of long COVID treatment can further contribute to a poorer quality of life. Long-term COVID health services are evolving, but there are no randomized trials about how to improve patient quality of life. Specialized centers have been established in some countries, and there has been a worldwide call for the development of rehabilitation programs and services for long COVID patients (53).

Several other studies have confirmed a lower quality of life in patients with COVID-19 and after overcoming COVID-19 (31, 54, 55). Quality of life in patients after overcoming COVID-19 was evaluated in a study by Shah et al. (56), who used the EuroQol group five-dimensions to measure quality life. As in our study, a negative effect on quality of life was found in 81.1% of the patients who reported pain and discomfort, 79.5% of the problems with normal activities, 68.7% of anxiety and depression, and 56.2% and problems with mobility (*p* < 0.05).

In our study, a higher incidence of pain was observed compared to the healthy control group. Patients with long COVID can experience multiple pain conditions (1, 4, 14, 57). It is possible that there was bias in our study due to the ages of the patients compared to ages of the healthy control group. According to Yezierski et al. (58), increases in pain sensitivity under different experimental conditions may be explained by age-related anatomical, physiological, and biochemical changes, resulting in compensatory changes in homeostatic mechanisms and the intrinsic plasticity of somatosensory pathways involved in the processing and perception of pain.

The main limitation of this study is that the research was entirely based on data collection using an electronic questionnaire, without clinical, psychological, or biological evaluations. Questionnaires are affected by significant selection bias, as only those patients who want to complete the questionnaires are included. The control group consisted of students of physiotherapy and physical education. This resulted in potential limitations due to the difference in age composition, and the assumption that younger students will have a better quality of life than an older control group. On the other hand, there are published studies showing that people around the age of 50 can have a better quality of life compared to older people. According to Netuveli et al. (59) quality of life increases from 50 years (CASP 19 score 44.4) to peak at 68 years (CASP-19 score. From there it gradually starts to decline. In our study, the mean age was 41.3 (±10.5). It is likely that at this age the quality of life may not be negatively affected due to age.

## Conclusions

A lower quality of life was found in patients with long COVID compared to the healthy control group. The low quality of life in patients with persistent symptoms after overcoming COVID-19 indicates a need for therapeutic intervention to improve the quality of life of patients.

## Data availability statement

The raw data supporting the conclusions of this article will be made available by the authors, without undue reservation.

## Ethics statement

The studies involving human participants were reviewed and approved by Ethics Committee of Matej Bel University under the number FF/123/2022. The patients/participants provided their written informed consent to participate in this study.

## Author contributions

DL, SD, AB, and EL: conceptualization and investigation. DL, LB, and PB: methodology and writing. AB and SD: validation and project administration. EL and DL: formal analysis and original draft preparation. All authors have read and agreed to the published version of the manuscript.

## Funding

This research was funded by the Ministry of Health, Czech Republic; Conceptual Development of Research Organization (FNBr, 65269705).

## Conflict of interest

The authors declare that the research was conducted in the absence of any commercial or financial relationships that could be construed as a potential conflict of interest.

## Publisher's note

All claims expressed in this article are solely those of the authors and do not necessarily represent those of their affiliated organizations, or those of the publisher, the editors and the reviewers. Any product that may be evaluated in this article, or claim that may be made by its manufacturer, is not guaranteed or endorsed by the publisher.
